# Implementation of a Booster Sexual Health Education Curriculum for Older Adolescents in Rural Communities

**DOI:** 10.1177/15248399231221156

**Published:** 2024-01-05

**Authors:** Nancy F. Berglas, Salish Harrison, Julio Romero, Natasha Borgen, Martha J. Decker

**Affiliations:** 1 University of California, San Francisco, Oakland, CA, USA; 2 University of California, San Francisco, San Francisco, CA, USA; 3 Fresno Economic Opportunities Commission, Fresno, CA, USA

**Keywords:** adolescents, sex education, sexual health, school health, mental health, youth voice

## Abstract

Ongoing education on sexual health and other health promotion topics is critical as young people transition into adulthood. A “booster” round of education may be an effective strategy to reinforce information previously taught and expand to additional topics relevant later in adolescence. In partnership with a Youth Advisory Council, we co-designed READY, Set, Go!, a booster curriculum for older adolescents with modules covering adult preparation skills, sexual identity, relationships, reproductive health, and mental health. From November 2021 to January 2023, we provided the curriculum to 21 cohorts of 12th grade students (*N* = 433) in rural communities of Fresno County, CA, and conducted an implementation evaluation to assess its feasibility in school settings, acceptability by participants, and changes in short-term outcomes. Health educators completed implementation logs to track program adaptations. Youth completed pretest/posttest surveys to assess changes in outcomes and participant satisfaction. We used descriptive statistics to examine program adaptations and satisfaction. We used multivariable regression models to examine changes in outcomes, adjusted for sociodemographic characteristics. Health educators completed most activities as planned, with adaptations occurring in response to youth needs and scheduling limitations. Sexual health knowledge, confidence in adult preparation skills, awareness of local sexual and mental health services, and willingness to seek health services all increased significantly from pretest to posttest. Youth feedback was strongly positive. We conclude that booster sexual health education is a promising strategy to address critical knowledge gaps and support health promotion, especially in rural and other under-resourced communities.

Access to comprehensive sexual health information is critical for promoting the sexual health of young people as they transition into adulthood. In the United States, national sex education standards have identified the essential information and skills that adolescents should receive in school settings, advocating for an approach that is comprehensive in content and sequential in execution (Future of Sex Education Initiative, 2020). In practice, however, implementation is inconsistent, limited in hours, and often focused narrowly on pregnancy and disease prevention rather than a broader range of recommended sexual health topics, particularly those relevant for older adolescents (Kantor & Lindberg, 2020).

The most commonly used and evaluated school-based sexual health education curricula are designed for implementation during middle school and early high school (Goesling et al., 2014; U.S. Department of Health and Human Services, 2017). This timing comes at the recommendation of sexual health professionals to ensure that young people receive information prior to engaging in sexual activity (Coyle & Glassman, 2016; Goldman, 2011). Indeed, many programs have been shown to be effective at reducing sexual risk behaviors among adolescents who are not yet sexually active (Goldfarb & Lieberman, 2021; Mueller et al., 2008). However, this timing may also have drawbacks, namely that youth receive education at a young age without further attention as they progress through adolescence.

A supplemental “booster” education program later in high school may be an effective strategy to reinforce information previously taught and expand to cover additional topics relevant in later years (Berglas et al., 2023; Pedlow & Carey, 2004). Educational research on instructional strategies has demonstrated the positive effects of spaced repetition, which allows students to better retain and apply information as it is provided over time (Dunlosky et al., 2013; Kang, 2016). A booster sexual health education program that addresses sexual communication, negotiation, and decision-making may also be more salient for older adolescents as sexual activity naturally increases with age, with 19% of ninth grade students reporting having had sex compared with 57% of 12th grade students (Pedlow & Carey, 2004; U.S. Department of Health and Human Services, 2020). Providing education at later ages also offers the opportunity to adjust information and teaching strategies to match the substantial physical, cognitive, emotional, and social changes of adolescence. With these changes, older adolescents face a host of needs—in terms of their sexual health, as well as their overall well-being—that could be met through a booster program (Berglas et al., 2023). Despite this potential, the design, implementation, and evaluation of booster sexual health education is quite limited (Haberland et al., 2018).

The need for a booster program may be particularly relevant for youth growing up in rural communities. Rural youth are more likely to be sexually active (Daniels et al., 2017; Ng & Kaye, 2015), less likely to use contraception the first time they have sex (Ng & Kaye, 2015), and less likely to be aware of local sexual health services (Geske et al., 2016; Yarger et al., 2017). Despite these increased needs, youth in rural areas are less likely to report having received any formal sex education (Lindberg et al., 2016), and few sexual health education programs have been developed for or adapted to meet the needs of rural youth (Goesling et al., 2014).

The purpose of this study is to document the pilot implementation of the *READY, Set, Go!* curriculum, a booster sexual health education curriculum that was co-designed with adolescents. Specifically, we evaluate the feasibility and acceptability of the curriculum’s implementation in high schools in rural communities of Fresno County, California. Furthermore, we share these findings to encourage discussion of booster education as an innovative model to promote the health of youth as they transition into adulthood.

## Project Overview

The development of a new booster sexual health education curriculum was undertaken as part of the Rural Education and Development for Youth (READY) project, a federally funded initiative to meet the sexual health information and service needs of youth in rural, predominantly Latino communities of Fresno County, California. The READY project involved multiple components, including implementation of an evidence-based sexual health education curriculum for ninth grade students, workshops for parents/guardians, capacity building for school staff, and engagement with local health clinics to promote access to services. At the onset, researchers conducted focus groups with youth and key informant interviews with youth-serving professionals to understand the needs and existing sexual health education of youth living in the communities (Berglas et al., 2023). This formative work was initiated with a focus on sexual health, but open to learning about the broader needs of young people in these communities. Through thematic analyses, we identified a need for booster education toward the end of high school that reinforced existing sexual health knowledge; expanded beyond traditional areas of sexual health education to include content on healthy relationships, sexual identity, and mental health; and prepared youth for the transition to adulthood.

With this foundation, the READY team recruited a Youth Advisory Council, comprising young people from local communities, to support the development and evaluation of a new booster curriculum. From May to August 2021, the Youth Advisory Council met to discuss and develop program content and activities. The Youth Advisory Council included 11 male and female youth, ages 14 to 18, from three communities. Curriculum development was facilitated by two health educators, a member of the research team with past experience as a health educator, and a consultant with expertise in sexual health education and curriculum development. Over more than 20 hours at 11 meetings, the Youth Advisory Council brainstormed potential ideas and topics, ranked the most important topics, developed modules and learning objectives, and tested activities.

The new curriculum—named *READY, Set, Go!*—consists of five modules that address preparation for adulthood, sexual identity, relationships, reproductive health, and mental health and self-advocacy (Table 1). Each module included learning objectives that outline the major concepts youth are expected to learn, facilitation notes with instructions on how to implement the curriculum effectively, scripted text for key content, presentation slides, and activities including worksheets, videos, and discussion topics. Modules were designed to be implemented in a 50-minute class period. In addition, a resource website was created to provide access to key resources aligned with each module, as well as information for parents and guardians. Health educators piloted the modules with the Youth Advisory Council and other small groups in community settings.

**Table 1:** Overview of *READY, Set, Go!* Curriculum Topics

Module	Content areas
1. Adult Preparation	• Educational and career options after high school • Basics of personal finance • Budgeting, saving, and building credit
2. Sexual Identity	• Components of sexual identity • Sources that influence ideas about sexual diversity • Effects of negative messages about sexual diversity • Supporting sexual diversity, being an ally
3. Relationships	• Communication styles • Affirmative consent • Healthy and unhealthy relationships • Abusive relationships
4. Reproductive Health	• STI and HIV prevention strategies • Contraceptive methods • Pregnancy options • Accessing sexual health services • Expectations about parenting
5. Mental Health and Well-Being	• Components and impacts of mental health • Factors that influence mental health • Warning signs of poor mental health • Healthy and unhealthy coping mechanisms

## Conceptual Framework

*READY, Set, Go!* was developed using the concept of “youth voice,” which recognizes that youth are the key stakeholders and experts in their own lives and educational needs. Youth voice engages and calls upon young people to name their own issues and concerns, “creating more meaningful experiences that help meet [their] fundamental developmental needs, especially for students who otherwise do not find meaning in their school sexuality education experiences” (Sanjakdar et al., 2015). The engagement of youth voice is integral to improving relevance and effectiveness (Corcoran et al., 2020), yet typically program content is defined by adults for youth, rather than with them (Fakoya et al., 2022). The related frameworks of youth-centered design and youth-led participatory action research similarly argue for the importance of engaging youth as partners in design, implementation, and evaluation (Fakoya et al., 2022; Ozer, 2016).

## Method

### Study Design

*READY, Set, Go!* was implemented with primarily 12th grade students attending four high schools in three rural communities of Fresno County between November 2021 and January 2023. We designed an implementation evaluation to assess program fidelity, short-term changes in knowledge and skills, and participant satisfaction. The institutional review board of the University of California, San Francisco approved the study protocol.

### Data Collection

To assess fidelity of implementation, we developed a structured log that asked health educators to report on curriculum delivery for every cohort. The implementation log asked educators to report on the predefined activities for each of the five modules, reporting on adaptations. Within each module, educators reported whether each activity was completed as planned, included unplanned adaptations, or was not conducted. In addition to these quantitative items, educators were provided space to elaborate on each activity, module, and cohort if they chose. Implementation logs were collected for 20 of 21 cohorts.

To track youth outcomes, we developed a brief pretest/posttest survey to be completed on the first and last days of class. Participants completed surveys using their own smart phones to access a web-based link through Qualtrics, a secure platform for data encryption and transmission. Paper surveys were available as needed. Surveys were available in English and Spanish and took 10 to 15 minutes to complete. The survey began with an information sheet detailing the study purpose, risks, and benefits; participants gave assent by clicking through to the survey questions. The California Education Code does not require active parental consent for minors to participate in the evaluation of school-based sexual health education programs.

On the pretest survey, youth were asked to report sociodemographic characteristics, including age, race/ethnicity, languages spoken at home, gender identity, sexual orientation, and socioeconomic status (Goodman et al., 2001). On the pretest and posttest surveys, youth were asked to respond to short-term outcomes measures. Confidence in adult preparation skills was assessed using nine items, including writing a resume, making a budget, managing time well, and managing stress. Responses ranged from 1 (*not at all confident*) to 4 (*very confident*) and were averaged to create a mean scale. Sexual health knowledge was assessed using eight true/false items about healthy relationships, STI/HIV prevention and transmission, and pregnancy prevention. The number of correct responses was summed as an index ranging from 0 to 8, with a higher score reflecting greater knowledge. Two dichotomous items assessed awareness of local services, asking youth whether they knew of a place they could get “sexual health services (like birth control, pregnancy tests, and STI/HIV tests)” and “counseling or other mental health services.” Two items assessed willingness to seek sexual health and mental health services if needed. Responses ranged from 1 (*definitely no*) to 4 (*definitely yes*) and averaged to create a mean scale. Finally, on the posttest survey, youth were asked three questions indicating whether they found the program interesting (very/somewhat/not very), found the content useful (very/somewhat/not very), and felt comfortable asking questions (yes/no).

### Analysis

For implementation logs, we used descriptive statistics to examine adaptations and the classroom environment. We summarized qualitative entries provided by educators and provide examples to further explain implementation successes and challenges.

For youth surveys, we examined baseline characteristics using descriptive statistics for the entire sample. We matched pretest (*n* = 433) and posttest (*n* = 353) surveys within each cohort using four self-reported identification questions (Grube et al., 1989). We assessed differences in sociodemographic characteristics between the overall and matched samples using bivariate analysis. In the matched sample, we assessed the unadjusted change using simple linear and logistic regression models for continuous and dichotomous outcomes, respectively. We then used multivariable linear and logistic regression models to examine change adjusted for sociodemographic characteristics (age, gender identity, sexual orientation, socioeconomic status); we did not control for race/ethnicity, due to lack of variation in the sample. In all models, we clustered standard errors to account for nonindependence of responses. Finally, we used descriptive statistics to report on program satisfaction.

## Results

### Implementation and Adaptations

*READY, Set, Go!* was implemented with 21 cohorts (mean 21, range 10–31 participants) in four high schools from November 2021 through January 2023. Twenty cohorts were implemented during the school day, and one as an after-school program on the school campus. Based on pretest surveys (*n* = 433), nearly all youth (97%) were in 12th grade, with a mean age of 17.2 years (Table 2). Nearly all (96%) identified as Hispanic, and 80% spoke Spanish at home. Fifty-two percent of youth identified as male, 46% as female, and 2% as trans, nonbinary, or other gender. Eighty-six percent reported their sexual orientation as straight. There were no statistically significant differences in any sociodemographic characteristics between the overall sample and matched analytic sample (*n* = 268).

**Table 2:** Description of Youth Survey Sample

Characteristic	Pretest sample (n = 432)		Matched sample^a^ (n = 268)	
Age in years				
15 or 16	21	5%	13	5%
17	281	65%	164	68%
18 or older	130	30%	63	26%
Mean age (*M*, *SD*)	17.2	(*SD* 0.6)	17.2	(*SD* 0.5)
Grade				
10th/11th grade	11	2%	5	2%
12th grade	406	98%	225	98%
Race/ethnicity				
Hispanic	416	96%	233	97%
Non-Hispanic Black	1	<1%	0	0%
Non-Hispanic White	9	2%	7	3%
Non-Hispanic Other	6	1%	1	0%
Gender				
Female	199	46%	123	51%
Male	224	52%	113	47%
Trans/nonbinary/Other	10	2%	5	1%
Sexual orientation				
Straight/heterosexual	364	86%	199	84%
LGBTQ	60	14%	37	16%
Languages spoken at home^b^				
English	251	58%	143	53%
Spanish	346	80%	199	74%
Social status scale (*M*, *SD*)	5.6	(*SD* 1.8)	5.6	(*SD* 1.7)

a There were no statistically significant differences between the pretest and matched samples for any sociodemographic variable.

b Youth may have reported more than one language spoken at home.

Table 3 displays the percent of activities that were implemented as planned for each module. Unplanned adaptations varied by module, including 24% of activities in the reproductive health module, 20% of adult preparation activities, 18% of activities in the mental health module, and 15% of relationship activities. These adaptations reflected changes deemed necessary by the health educator in real time in response to youth’s needs, questions, and familiarity with content. For example, based on educator reports, financial aid was not covered at one school because the 12th grade had already participated in a college readiness program. Other changes were due to time constraints, indicating a need to cut down material in the curriculum to fit the school setting. Health educators adapted scenario-based activities (e.g., on contraception and parenting), choosing to use only a couple of scenarios as a large group, rather than breaking into pairs. In addition, changes to school bell schedules, morning announcements, and sports rallies were named as reasons why activities were interrupted or shortened.

**Table 3:** Percent of *READY, Set, Go!* Activities Implemented as Planned, by Module (*N* = 20 Cohorts)

Module	% of activities implemented as planned	% of activities with unplanned adaptations	% of activities not implemented
Adult Preparation	80	20	0
Sexual Identity	5	0	95
Relationships	84	15	1
Reproductive Health	74	24	2
Mental Health and Well-Being	80	18	3

For nearly all cohorts, school administrators gave health educators 4 days to implement the program during a holiday week, rather than a full 5-day week. As a result, health educators decided not to implement Module 2 on sexual identity. This module was the shortest timewise and was most adaptable at having key messages and themes incorporated throughout the other modules. For example, an activity from Module 2 on “Being an Ally” provided scenarios and asked youth to brainstorm ways to support their peers. These scenarios were incorporated into the final activity in Module 5 “Advocating for Yourself,” and the entire activity was reframed as “Advocating for Yourself and Others.” Key concepts of sexual identity were addressed during Modules 3 and 4 and were reinforced by use of gender-neutral language and diverse scenarios. In addition to being the most flexible module, community partners reported that schools were concerned about the subject matter of sexual identity, affecting the decision to drop this module.

### Changes in Short-Term Outcomes

Confidence in adult preparation skills increased from pretest to posttest (2.83–2.94); this change was statistically significant in models adjusted for sociodemographic characteristics (*b* = 0.70; 95% confidence interval [CI] = [0.49, 0.92]). Sexual health knowledge increased from 3.53 to 4.16 (*b* = 0.45; 95% CI = [0.14, 0.75]; see Table 4).

**Table 4:** Change in Short-Term Outcomes Before and After Participation in *READY, Set, Go!*

Continuous outcomes	M (SD) at pretest	M (SD) at posttest	Adjusted coefficient^a^	95% CI	p value
Confidence in adult preparation skills (1–4)	2.83 (0.59)	2.94 (0.58)	0.70	0.49–.0.92	.001
Sexual health knowledge (0–8)	3.53 (1.907)	4.16 (2.81)	0.45	0.14–0.75	.015
Willingness to seek sexual health services (1–4)	3.20 (0.69)	3.29 (0.75)	0.51	0.40–0.61	<.001
Willingness to seek mental health services (1–4)	2.81 (0.98)	3.04 (0.82)	0.51	0.42–0.61	<.001
*Dichotomous outcomes*	*% at pretest*	*% at posttest*	*Adjusted odds ratio* ^a^	*95% CI*	p *value*
Awareness of sexual health services	56	79	5.39	1.59–18.29	.007
Awareness of mental health services	59	86	9.22	2.77–30.68	<.001

a Adjusted linear and logistic regression models control for age, gender identity, sexual orientation, and socioeconomic status and include clustered standard errors by cohort.

At posttest, a greater proportion of youth reported awareness of a place to access sexual health services (56% vs. 79%); this change was statistically significant in adjusted models (aOR: 5.39; 95% CI = [1.59, 18.29]). Similarly, a greater proportion of youth reported awareness of a place to access mental health services (59% vs. 86%; aOR: 9.22; 95% CI = [2.77, 30.68]). The wide confidence intervals indicate some instability in the results of these two outcomes.

Willingness to seek sexual health services increased from pretest to posttest (3.20 vs. 3.29); this change was statistically significant in adjusted models (*b* = 0.51; 95% CI = [0.40, 0.61]). Similarly, willingness to seek mental health services increased from 2.81 versus 3.04 (*b* = 0.51; 95% CI = [0.42, 0.61]).

### Program Satisfaction

Overall, youth responded positively to the program. Two thirds (67%) found the program to be very interesting, and an additional 29% found it to be somewhat interesting. Eighty-four percent found the program content to very useful, and an additional 15% found it to be somewhat useful. Three quarters (75%) said they were comfortable asking questions.

## Discussion

The implementation of the *READY, Set, Go!* curriculum was largely successful, indicating feasibility in school settings, acceptability by youth participants, and positive short-term changes in outcomes. Health educators completed most activities as planned, and unplanned adaptations were mostly related to adjustments that responded to the needs of the particular audience or scheduling limitations. Participant feedback about the program was positive. From the beginning to end of the brief curriculum, youth reported increased confidence in their adult preparedness skills, knowledge of sexual health, awareness of sexual and mental health services, and willingness to seek out such services if needed.

A hallmark of *READY, Set, Go!* was its inclusion and centering of youth voice in its development. Recent research has called out youth-centered design as critical to successful program development (Fakoya et al., 2022), yet it still quite rare to include youth in the formative phases of curriculum development or even ask youth about gaps or preferences in their educational content. Engaging rural youth and youth of color is particularly rare (Bañales et al., 2023; Harris et al., 2021). Partnering with young people in curriculum development is invaluable to ensuring that programs are reflective of their needs, assets, and values (Corcoran et al., 2020; Fakoya et al., 2022). The implementation of *READY, Set, Go!* represents the culmination of the input of youth in our focus groups and, especially, the Youth Advisory Council. Their voices motivated the development of a curriculum that augments traditional sexual health education content and expands to address topics of importance to young people today, most notably healthy relationships, gender identity and sexual orientation, adult preparedness, and mental health. For example, the inclusion of adult preparedness and mental health may be considered outside the domain of a typical sexual health education program, but was highlighted as critical by both youth and youth-serving professionals in our formative work (Berglas et al., 2023). These insights are reflected in the positive feedback of *READY, Set, Go!* participants, who found the new curriculum to be interesting and relevant. Additional research will need to understand how best to integrate these topics or determine whether separating course content is more effective.

The implementation of *READY, Set, Go!* was challenged by the inability to implement all five modules consistently in school settings; specifically, the sexual identity module was consistently cut when school availability was limited to 4 days. The pressures of academic requirements, limited resources, and local politics faced by schools are well known. However, schools are a natural place for promoting the health of youth, especially in rural communities where schools often serve as a hub for community education and supportive services (Berglas et al., 2023). Given this context, the inability to consistently implement the inclusivity module raises critical questions of how and where to respond to youth-expressed needs if schools are not willing or able to do so.

We note this study’s limitations. Because this is the first evaluation of a new curriculum, it was purposefully designed to focus on feasibility, acceptability, and short-term changes in knowledge. The study design did not include a comparison group or additional follow-up surveys to determine whether changes in outcomes persisted or affected behaviors. Future studies will need to incorporate validated survey measures and qualitative data to rigorously understand changes in youth’s knowledge, attitudes, skills, and behaviors. Although the survey included few sensitive personal questions, misreporting or omissions may have affected results. Challenges faced in the matching of pretest/posttest surveys and the resulting exclusion of unmatched surveys may have affected results, especially if youth who did not complete both surveys were less satisfied with the program or were less likely to experience change in outcomes; however, we note the lack of significant sociodemographic differences between the overall and matched samples. As discussed, we were unable to implement the module on sexual and gender identity in many schools, and therefore the curriculum as a whole has not been assessed. An expanded implementation and more rigorous evaluation is necessary to determine which findings will be reliable and whether there will be evidence of long-term impact.

### Implications for Practice, Policy, and Research

In addition to highlighting specific areas of promise and potential for improvement of the *READY, Set, Go!* curriculum, this study has implications for health promotion more broadly. Policymakers, school districts, and other youth-serving organizations should consider implementing sequential, spaced sexual health education programs to meet ongoing needs, reflect natural developmental changes, and promote positive health outcomes. The booster model may need to be inherently flexible to fit the needs and contexts of different communities—in terms of the sexual health education that has previously been provided, the availability of other school programs and community resources, and the emerging needs of youth. Youth in rural communities may face distinct gaps in access to knowledge and resources that require further support. Authentically engaging rural youth is therefore crucial in program development and curricular adaptations to respond to their distinct needs, interests, and priorities. Further research is warranted on this promising model, including its effectiveness with different populations, the utility of integrating additional content such as mental health into sexual health education, and how booster education fits with existing standards of practice.

## Conclusion

Booster sexual health education is a promising strategy to provide developmentally appropriate information, address critical knowledge gaps, and support health promotion for older adolescents. The development of *READY, Set, Go!* highlights the need for meaningful engagement of youth in all stages of program development, implementation, and evaluation.
